# From field courses to DNA barcoding data release for West Papua - making specimens and identifications from university courses more sustainable

**DOI:** 10.3897/BDJ.6.e25237

**Published:** 2018-06-05

**Authors:** Bruno Cancian de Araujo, Stefan Schmidt, Olga Schmidt, Thomas von Rintelen, Agustinus Kilmaskossu, Rawati Panjaitan, Michael Balke

**Affiliations:** 1 SNSB-Zoologische Staatssammlung München, Munich, Germany; 2 Museum für Naturkunde, Leibniz-Institut für Evolutions- und Biodiversitätsforschung, Berlin, Germany; 3 Department of Biology, Faculty of Sciences and Mathematics, State University of Papua (UNIPA), Jalan Gunung Salju Amban, Manokwari, Indonesia

## Abstract

The diversity of insects collected during entomological field courses at the University of West Papua (UNIPA), Indonesia, is studied using DNA barcoding tools. The results were compared with public data available for West Papua in the Barcode of Life Data System. During two training courses in 2013 and 2015, 1,052 specimens of insects were collected at eight sites near Manokwari in northern West Papua. The DNA sequences obtained from these specimens were assigned to 311 Barcode Index Numbers (BINs) and represent species in 27 families of Lepidoptera, Hymenoptera and Coleoptera. Of those BINs, 294 (95%) were new to West Papua. The study suggests that DNA barcoding applied to university courses achieves several goals, including capacity building and hands-on experience in molecular biodiversity assessment. In addition, it can provide valuable biodiversity data that are globally available to researchers for further studies.

## Introduction

Carrying out field courses for students is a central aspect of any capacity building effort at universities, including and perhaps especially so in tropical countries (see Basset et al. 2000, Basset et al. 2004, Novotny et al. 2012, Tänzler et al. 2012, Rintelen et al. 2017). They serve mainly the purpose of demonstrating sampling, preparation and identification methods for insects and other arthropods. The scientific value of insect collecting during student courses can be dramatically increased if the specimens are adequately preserved and/or mounted for long-term storage in a scientific collection (Lopez-Vaamonde et al. 2012, Schmidt et al. 2017). This applies even more if samples are then utilised for more sustainable methods of biodiversity assessment, such as DNA barcode analysis and if sequence and associated collecting data are generally accessible for researchers nationally and internationally (see Hebert et al. 2003, Hajibabaei et al. 2005, Janzen et al. 2009, Gwiazdowski et al. 2015, Wirta et al. 2015, Miller et al. 2016).

The present study makes an attempt to use specimens of insects collected during student courses in a more sustainable way. In particular, we tried to maximise the potential benefit of field and entomological training courses at the University of West Papua (UNIPA) on the western part of the island of New Guinea. The courses were conducted by a team of entomologists from the Zoologische Staatssammlung (ZSM) in Munich, Germany, which was followed up by a repeated staff exchange from UNIPA to the ZSM.

## Material and Methods

The methods were described in Cancian de Araujo et al. (2017), with the following differences:

### Specimen collecting and processing

In 2013 and 2015, 1,052 specimens of insects were collected during capacity building courses by lecturers and students of the State University of Papua (UNIPA) in Manokwari. The collections mainly served as a vehicle to demonstrate field survey methods and subsequent laboratory procedures for sustainable biodiversity inventory and discovery. Targeted field work was coordinated by RP and AK and part of the laboratory work was conducted by RP during her stay at the SNSB-Zoologische Staatssammlung München (ZSM, Bavarian State Collection of Zoology) in 2014 under the supervision of MB, SS and OS.

**Figure 1.**
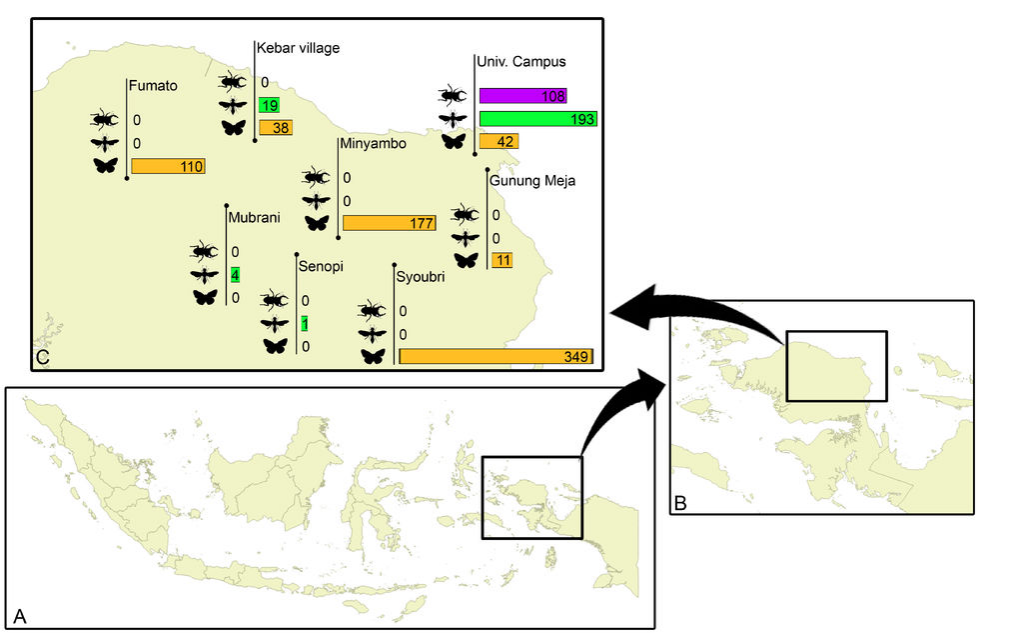
Specimens distribution per site and Order.

Samples were collected at eight sites in the Indonesian province West Papua, viz. Fumato, Kebar Village, Minyambo, Mubrani, Syoubri, Senopi, Gunung Meja and the Papua University Campus in Manokwari (Fig. 1). The latter was a short Malaise trapping exercise with one trap that was operated for three weeks. For more details about the field and lab protocols see Schmidt et al. (2015), Schmidt et al. (2017) and below. The specimen data are accessible on BOLD through the following doi: 10.5883/DS-INWPAPUA and through GenBank (Accession nos MH094885-MH095566),

### Data acquisition

The specimen data and result files generated for the present study were downloaded directly from the Barcode of Life Data Systems (BOLD, http://www.boldsystems.org) workbench. In addition, all other public records from the province West Papua and other Indonesian areas from the western half of the island of New Guinea present in BOLD were obtained through the REST API of the BOLD platform on 31-Jan-2018. We applied the “Full Data Retrieval” parameters geo=Papua|West%20Papua|Papua%20Barat and marker=COI–5P in order to gather all public records from West Papua with the standard DNA barcoding marker (COI–5P).

### Data processing

The files that were downloaded contained information on each record including the Barcode Index Number (BIN), collection data and taxonomy. The data were evaluated in terms of BIN diversity, spatial distribution of specimens, taxonomic identification depth and taxonomic diversity. The results were compared in terms of diversity of BINs, exclusive and shared BINs and BIN distribution. Analyses and comparisons were made using Microsoft Excel. The number of BINs shared by the two sources was evaluated and after that, the shared BINs were subtracted from our West Papua list in order to highlight the contribution of our case study for West Papuan records in general. The map with collecting records was created using Quantum GIS (vers. 2.8).

## Results

**Figure 2.**
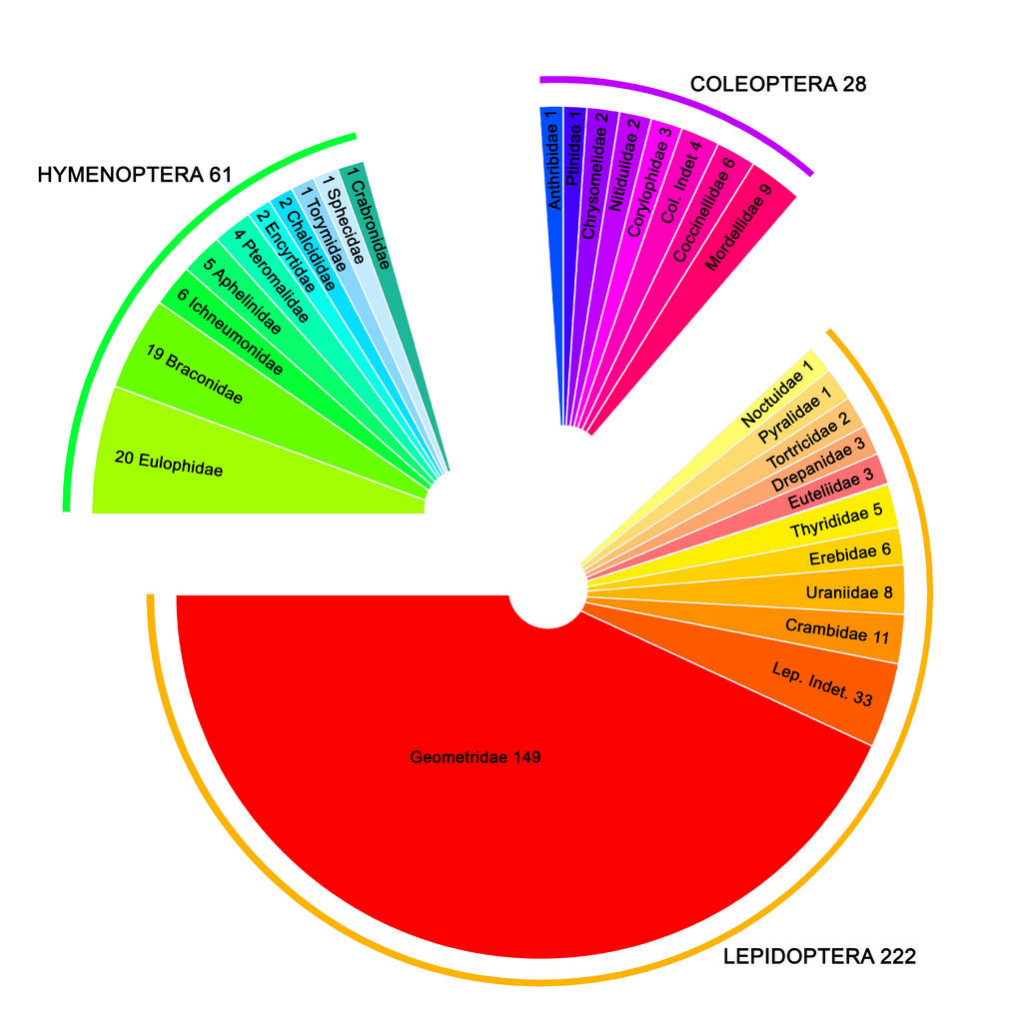
BINs distribution per Order and Family.

Between 2013 and 2015, 1,052 specimens from West Papua were processed. The records are distributed in six areas in West Papua and were collected at altitudes between 80 and 1,555 meters above sea level. The taxa belong to three insect orders: Coleoptera (108), Hymenoptera (217) and Lepidoptera (727). The geographic distribution per site and taxon are presented in Fig. 1. Out of these 1,052 specimens, the CO1-5P barcode sequence was recovered from 686 specimens, corresponding to 311 BINs from at least 27 families of insects (Fig. 2).

When searching for public data of arthropods from West Papua in BOLD, we recovered 1,268 records that were assigned to 584 BINs. The records belong to 10 orders with the most common being Lepidoptera (910 records, 441 BINs), Coleoptera (214 records, 68 BINs) and Decapoda (60 records, 30 BINs). When comparing our records with the public data available on BOLD, only 17 BINs (5%) had been recorded before from West Papua, whereas 294 BINs (95%) were new records for this area in BOLD.

A comparison of BINs recorded in our study that were also recorded from elsewhere showed that 74 BINs (24%) were already present in BOLD from regions outside of West Papua, mainly from Australia (39 BINs), Papua New Guinea (23 BINs), French Polynesia (6 BINs) and Indonesian locations other than West Papua (23 BINs) (Table 1). The species is given if the BIN was associated with a species (or genus) name in BOLD, but it should be stressed that, for many species, the barcode-based species level identification require verification based on morphology by a specialist.

Two taxa, *Spoladea recurvalis* (Lepidoptera, Crambidae) and a species of *Cotesia* (Hymenoptera, Braconidae), had a wider distribution with records from five or more countries (Table 1). The remaining 237 BINs (76%) were recorded excusively from West Papua.

## Discussion

The high number of BINs that are exclusive to an area with comparatively well studied surroundings, highlights the urgency of studying the biodiversity of tropical regions. Analysis of 1,052 specimens increased the diversity of known species in this particular area 1.5-fold, from 583 to 877 species, as expressed by BINs that have been shown to closely relate to biological species. This was achieved by analysing a handful of randomly collected samples obtained by students during field courses under the supervision of entomologists from the ZSM (Munich).

Even for well studied groups like Lepidoptera, the study led to an increase of 205 BINs, corresponding to nearly one third (32%) of all species known so far for this insect order from West Papua. For less well known groups like Hymenoptera and Coleoptera, all BINs were new to West Papua. It is important to stress that nearly all Coleoptera and Hymenoptera specimens of the present study were collected at one site, the campus of the Papua University at Manokwari, showing the potential for a significantly higher number of discoveries with a broader sampling regime across different elevations and actually investigating primary forest areas. The Geometridae (Lepidoptera) was a target group for collecting which explains the predominance of geometrid moths, representing nearly half (48%) of the BINs.

Our study suggests that DNA barcoding applied to university courses achieves several goals, including capacity building, hands-on experience in molecular biodiversity assessment and it provides valuable data that are globally available by researchers for further studies (see also Vernooy et al. 2010). Specimens that would usually only be identified to order or perhaps genus level (and then often forgotten) can now serve to provide data in a sustainable manner. The data have become a community resource and are available for local researchers to benefit their research. Ideally, the next steps would include more focussed and specific project orientated field- and laboratory work that could strongly support the analysis of large scale patterns of diversity as outlined by Tänzler et al. (2012).

In a very similar context, DNA barcoding applied to samples obtained through activities of citizen scientists in remote localities (Janzen and Hallwachs 2011, Miller et al. 2014, Wilson et al. 2015, Jisming-See et al. 2016, Loos et al. 2015, Schilthuizen et al. 2017, Suprayitno et al. 2017, Freitag et al. 2018) could make significant, objective contributions to our understanding of the patterns of global biodiversity.

Finally, this simple experiment provided additional occurrence records for virtually cosmopolitan species like the Lepidoptera, Crambidae: *Spoladea recurvalis* and a widespread species of braconid wasps (Hymenoptera, Braconidae, *Cotesia* sp.), confirming the usefulness of DNA barcoding for the large scale assessment of global distribution patterns and also for monitoring the distribution and spread of invasive species.

## Figures and Tables

**Table 1.** Taxa with BOLD records from locations outside West Papua. Given are insect order, family, Barcode Index Number (BIN) and species (or genus) in case of a BOLD BIN match and country from where the species was recorded.

**Order**	**Family**	**BIN / Species**	**Country**
Coleoptera	Chrysomelidae	BOLD:ADG9196	Indonesia (West Sumatera)
Coleoptera	Coccinellidae	BOLD:AAH3306 (*Scymnus mitior*)	Australia
Coleoptera	Coccinellidae	BOLD:ADC0638	Indonesia (West Sumatera)
Coleoptera	Mordellidae	BOLD:ADC2466	Malaysia
Coleoptera	Nitidulidae	BOLD:ADG5133 (*Epuraea ocularis*)	Indonesia (West Sumatera), French Polynesia
Hymenoptera	Aphelinidae	BOLD:ADD0593	Indonesia (West Java)
Hymenoptera	Braconidae	BOLD:AAH1084 (*Cotesia* sp.)	Australia, French Polynesia, Indonesia (West Sumatera), Pakistan, Papua New Guinea, United Arab Emirates
Hymenoptera	Braconidae	BOLD:AAH1349	Indonesia (West Java), South Korea
Hymenoptera	Braconidae	BOLD:ADJ6741	Indonesia (West Java)
Hymenoptera	Chalcididae	BOLD:AAW0748	Australia
Hymenoptera	Crabronidae	BOLD:ACV0318	Indonesia (West Sumatera)
Hymenoptera	Encyrtidae	BOLD:ACP0359	Indonesia (Bali, West Sumatera), Australia
Hymenoptera	Eulophidae	BOLD:ADE0821	Indonesia (West Java)
Hymenoptera	Ichneumonidae	BOLD:AAH2022 (*Enicospilus* sp.)	Australia, Indonesia (West Sumatera)
Hymenoptera	Ichneumonidae	BOLD:ADG2435	Indonesia (West Java)
Hymenoptera	Ichneumonidae	BOLD:ADK3119	Indonesia (West Java)
Hymenoptera	Sphecidae	BOLD:AAH3486	Australia
Lepidoptera	Crambidae	BOLD:AAA3666 (*Spoladea recurvalis*)	Australia, Canada, China, French Polynesia, Israel, Japan, Pakistan, Seychelles, South Africa, South Korea, United States
Lepidoptera	Crambidae	BOLD:AAB5972 (*Prophantis adusta*)	Australia, Papua New Guinea
Lepidoptera	Crambidae	BOLD:AAC4723 (*Cnaphalocrocis poeyalis*)	Australia, French Polynesia
Lepidoptera	Crambidae	BOLD:AAD1174 (*Eurrhyparodes bracteolalis*)	Australia
Lepidoptera	Crambidae	BOLD:AAD7675 (*Tabidia insanalis*)	Australia, Papua New Guinea
Lepidoptera	Crambidae	BOLD:AAE0808 (*Agrioglypta eurytusalis*)	French Polynesia, Australia, Indonesia (West Java)
Lepidoptera	Crambidae	BOLD:AAE5257 (*Herpetogramma hipponalis*)	Australia
Lepidoptera	Crambidae	BOLD:AAI4942 (*Palpita uedai*)	Australia
Lepidoptera	Crambidae	BOLD:AAL8459 (*Parotis* sp.)	Papua New Guinea
Lepidoptera	Crambidae	BOLD:ABU8109	Indonesia (West Sumatera)
Lepidoptera	Drepanidae	BOLD:AAD3754 (*Tridrepana lunulata*)	Australia
Lepidoptera	Erebidae	BOLD:AAB3896 (Cyme nr pyraula)	Australia
Lepidoptera	Erebidae	BOLD:AAF0763 (*Polypogon fractalis*)	Australia
Lepidoptera	Erebidae	BOLD:AAZ1754 (*Harita nodyna*)	Australia
Lepidoptera	Erebidae	BOLD:ACE6049 (*Pogonia umbrifera*)	Australia
Lepidoptera	Euteliidae	BOLD:AAK0923 (*Anigraea cinctipalpis*)	Australia, Malaysia
Lepidoptera	Euteliidae	BOLD:ABA5144 (*Anigraea deleta*)	Malaysia
Lepidoptera	Geometridae	BOLD:AAA9837 (*Eucyclodes pieroides*)	Australia
Lepidoptera	Geometridae	BOLD:AAA9900 (*Hyposidra talaca*)	Indonesia (West Java), Australia, China, Papua New Guinea
Lepidoptera	Geometridae	BOLD:AAB0565 (*Idaea simplex*)	Australia
Lepidoptera	Geometridae	BOLD:AAB1570 (*Comostola leucomerata*)	Australia
Lepidoptera	Geometridae	BOLD:AAB8518	Australia, French Polynesia
Lepidoptera	Geometridae	BOLD:AAC1659 (*Pingasa chlora*)	Papua New Guinea
Lepidoptera	Geometridae	BOLD:AAD4641 (*Symmacra ochrea* or *S. solidaria*)	Papua New Guinea
Lepidoptera	Geometridae	BOLD:AAE2192 (*Chloroclystis cissocosma*)	Indonesia (West Java, East Java), Australia
Lepidoptera	Geometridae	BOLD:AAE7089 (*Agathiopsis basipuncta*)	Australia
Lepidoptera	Geometridae	BOLD:AAF3254 (*Krananda extranotata*)	Australia
Lepidoptera	Geometridae	BOLD:AAF9464	Papua New Guinea
Lepidoptera	Geometridae	BOLD:AAF9570 (*Episothalma obscurata*)	Australia
Lepidoptera	Geometridae	BOLD:AAF9586 (*Aeolochroma* sp.)	Papua New Guinea
Lepidoptera	Geometridae	BOLD:AAF9622	Indonesia (West Java, East Java)
Lepidoptera	Geometridae	BOLD:AAI6447	Australia
Lepidoptera	Geometridae	BOLD:AAI7614 (*Idaea elaphrodes*)	Australia
Lepidoptera	Geometridae	BOLD:AAL8324 (*Thalassodes* sp.)	Papua New Guinea
Lepidoptera	Geometridae	BOLD:AAR3997 (*Chloroclystis semiscripta*)	Indonesia (West Java)
Lepidoptera	Geometridae	BOLD:ABW8597 (*Paradromulia rufibrunnea*)	Papua New Guinea
Lepidoptera	Geometridae	BOLD:ABX5389 (*Cleora illustraria* or *C. repetita*)	Australia, Papua New Guinea
Lepidoptera	Geometridae	BOLD:ABX6387 (*Craspedosis aurigutta*)	Papua New Guinea
Lepidoptera	Geometridae	BOLD:ABY7397 (*Aeolochroma* sp.)	Papua New Guinea
Lepidoptera	Geometridae	BOLD:ABZ2247 (*Iridobapta argostola*)	Australia
Lepidoptera	Geometridae	BOLD:ACB0570 (*Dioscore meeki*)	Papua New Guinea
Lepidoptera	Geometridae	BOLD:ACB8931 (*Gymnoscelis* sp.)	Papua New Guinea
Lepidoptera	Geometridae	BOLD:ACE7174 (*Nadagarodes duplicipuncta* or *N. mysolata*)	Australia
Lepidoptera	Geometridae	BOLD:ACK8229 (*Thalassodes umbrimedia*)	Papua New Guinea
Lepidoptera	Geometridae	BOLD:ACM4629	Papua New Guinea
Lepidoptera	Geometridae	BOLD:ACP9799	Papua New Guinea
Lepidoptera	Geometridae	BOLD:ACZ0473	Indonesia (East Java)
Lepidoptera	Geometridae	BOLD:ACZ0547	Indonesia (Bali)
Lepidoptera	Geometridae	BOLD:ACZ0867	Indonesia (West Java)
Lepidoptera	Geometridae	BOLD:ACZ1181	Indonesia (West Java)
Lepidoptera	Noctuidae	BOLD:AAO8880 (*Argyrolepidia thoracophora*)	Australia
Lepidoptera	Thyrididae	BOLD:AAA8776 (*Mellea ordinaria* species complex)	Papua New Guinea
Lepidoptera	Thyrididae	BOLD:AAG6033 (*Canaea hyalospila* or *C. rusticata*)	Australia, Papua New Guinea
Lepidoptera	Tortricidae	BOLD:AAA9084 (*Adoxophyes templana* species comples)	Australia, Papua New Guinea
Lepidoptera	Tortricidae	BOLD:AAB4056 (*Homona trachyptera*)	Australia, Papua New Guinea
Lepidoptera	Uraniidae	BOLD:AAC9235 (*Cathetus euthysticha*)	Australia
Lepidoptera	Uraniidae	BOLD:AAD1023 (*Phazaca mutans*)	Australia

## References

[B4206655] Basset Y., Nonotny V., Miller S. E., Pyle R. (2000). Quantifying biodiversity: experiences with parataxonomists and digital photography in New Guinea and Guyana. Bio Science.

[B4206812] Basset Y., Novotny V., Miller S. E., Weiblen G. D., Missa O., Stewart J. A. (2004). Conservation and Biological Monitoring of Tropical Forests: The Role of Parataxonomists. Journal of Applied Ecology.

[B4202809] Cancian de Araujo Bruno, Schmidt Stefan, von Rintelen Thomas, Sutrisno Hari, von Rintelen Kristina, Ubaidillah Rosichon, Häuser Christoph, Peggie Djunijanti, Narakusumo R. P., Balke Michael (2017). IndoBioSys - DNA barcoding as a tool for the rapid assessment of hyperdiverse insect taxa in Indonesia: a status report. Treubia.

[B4283535] Freitag H., Pangantihon C., Njunjić I. (2018). Three new species of *Grouvellinus* Champion 1923 (Insecta, Coleoptera, Elmidae) from Maliau Basin, Sabah, Borneo, discovered by citizen scientists during the first Taxon Expedition. ZooKeys.

[B4242390] Gwiazdowski Rodger A., Foottit Robert G., L. Maw H. Eric, Hebert Paul D. N. (2015). The Hemiptera (Insecta) of Canada: Constructing a reference library of DNA barcodes. PLOS ONE.

[B4207391] Hajibabaei M., deWaard J. R., Ivanova N. V., Ratnasingham S., Dooh R. T., Kirk S. L., Mackie P. M., Hebert P. D. N. (2005). Critical factors for assembling a high volume of DNA barcodes. Philosophical Transactions of the Royal Society B: Biological Sciences.

[B4207371] Hebert P. D. N., Cywinska A., Ball S. L., deWaard J. R. (2003). Biological identifications through DNA barcodes. Proceedings of the Royal Society B: Biological Sciences.

[B4270342] Janzen Daniel H., Hallwachs Winnie (2011). Joining inventory by parataxonomists with DNA barcoding of a large complex tropical conserved wildland in Northwestern Costa Rica. PLoS ONE.

[B4294994] Janzen D. H., Hallwachs Winnie, Blandin Patrick, Burns J. M., Cadiou Jean-Marie, Chacon Isidro, Dapkey Tanya, Deans A. R., Epstein M. E., Espinoza Bernardo, Franclemont J. G., Haber W. A., Hajibabaei Mehrdad, Hall J. P. W., Hebert P. D. N., Gauld I. D., Harvey D. J., Hausmann Axel, Kitching I. J., Lafontaine D., Landry J. F., Lemaire Claude, Miller J. Y., Miller J. S., Miller Lee, Miller S. E, Montero J., Munroe E., Green S. R., Ratnasingham S., Rawlins J. E., Robbins R. K., Rodriguez J. J., Rougerie R., Sharkey M. J., Smith M. A., Solis M. A., Sullivan J. B., Thiacourt Paul, Wahl D. B., Weller S. J., Whitfield J. B., Willmott K. R., Wood D. M., Woodley N. E., Wilson J. J. (2009). Integration of DNA barcoding into an ongoing inventory of complex tropical biodiversity. Molecular Ecology Resources.

[B4266457] Jisming-See Shi-Wei, Sing Kong-Wah, Wilson John-James (2016). DNA barcodes and citizen science provoke a diversity reappraisal for the “ring” butterflies of Peninsular Malaysia (Ypthima: Satyrinae: Nymphalidae: Lepidoptera). Genome.

[B4266480] Loos Jacqueline, Horcea-Milcu A. I., Kirkland Paul, Hartel Tibor, Osváth-Ferencz Márta, Fischer Joern (2015). Challenges for biodiversity monitoring using citizen science in transitioning social–ecological systems. Journal for Nature Conservation.

[B4208082] Lopez-Vaamonde Carlos, Breman Floris C., Lees David C., Houdt Jeroen VAN, Prins Jurate DE (2012). Analysis of tissue dependent DNA yield for optimal sampling of micro-moths in large-scale biodiversity surveys. European Journal of Entomology.

[B4266417] Miller Jeremy, Schilthuizen Menno, Burmester Jennie, van der Graaf L., Merckx Vincent, Jocqué Merlijn, Kessler Paul, Fayle Tom, Breeschoten Thijmen, Broeren Regi, Bouman Roderick, Chua W. J., Feijen Frida, Fermont Tanita, Groen Kevin, Groen Marvin, Kil Nicolaas, Laat H. de, Moerland Michelangelo, Moncoquet Carole, Panjang Elisa, Philip Amelia, Roca-Eriksen Rebecca, Rooduijn Bastiaan, Santen Marit van, Swakman Violet, Evans Meaghan, Evans Luke, Love Kieran, Joscelyne Sarah, Tober Anya, Wilson Hannah, Ambu Laurentius, Goossens Benoit (2014). Dispatch from the field: ecology of ground-web-building spiders with description of a new species (Araneae, Symphytognathidae). Biodiversity Data Journal.

[B4207516] Miller Scott E., Hausmann Axel, Hallwachs Winnie, Janzen Daniel H. (2016). Advancing taxonomy and bioinventories with DNA barcodes. Philosophical Transactions of the Royal Society B: Biological Sciences.

[B4206758] Novotny V., Weiblen G. D., Miller S. E., Basset Y., Lowman M. D., Schowalter T. D., Franklin J. F. (2012). The role of paraecologists in Twenty-first-century tropical forest research. Methods in forest canopy research.

[B4292813] Rintelen Kristina von, Arida Evy, Häuser Christoph (2017). A review of biodiversity-related issues and challenges in megadiverse Indonesia and other Southeast Asian countries. Research Ideas and Outcomes.

[B4241872] Schilthuizen Menno, Seip Lilian, Otani Sean, Suhaimi Jadda, Njunjić Iva (2017). Three new minute leaf litter beetles discovered by citizen scientists in Maliau Basin, Malaysian Borneo (Coleoptera: Leiodidae, Chrysomelidae). Biodiversity Data Journal.

[B4206603] Schmidt Olga, Hausmann Axel, de Araujo Bruno Cancian, Sutrisno Hari, Peggie Djunijanti, Schmidt Stefan (2017). A streamlined collecting and preparation protocol for DNA barcoding of Lepidoptera as part of large-scale rapid biodiversity assessment projects, exemplified by the Indonesian Biodiversity Discovery and Information System (IndoBioSys). Biodiversity Data Journal.

[B4202798] Schmidt Stefan, Schmid-Egger Christian, Morinière Jérôme, Haszprunar Gerhard, Hebert Paul D. N. (2015). DNA barcoding largely supports 250 years of classical taxonomy: identifications for Central European bees (Hymenoptera, Apoidea partim). Molecular Ecology Resources.

[B4242400] Suprayitno Nano, Narakusumo Raden Pramesa, Rintelen Thomas von, Hendrich Lars, Balke Michael (2017). Taxonomy and Biogeography without frontiers – WhatsApp, Facebook and smartphone digital photography let citizen scientists in more remote localities step out of the dark. Biodiversity Data Journal.

[B4206437] Tänzler Rene, Sagata Katayo, Surbakti Suriani, Balke Michael, Riedel Alexander (2012). DNA barcoding for community ecology - how to tackle a hyperdiverse, mostly undescribed Melanesian Fauna. PLoS ONE.

[B4287003] Vernooy Ronnie, Haribabu Ejnavarzala, Muller Manuel Ruiz, Vogel Joseph Henry, Hebert Paul D. N., Schindel David E., Shimura Junko, Singer Gregory A. C. (2010). Barcoding life to conserve biological diversity: beyond the taxonomic imperative. PLoS Biology.

[B4266467] Wilson John-James, Jisming-See Shi-Wei, Brandon-Mong Guo-Jie, Lim Aik-Hean, Lim Voon-Ching, Lee Ping-Shin, Sing Kong-Wah (2015). Citizen science: the first Peninsular Malaysia butterfly count. Biodiversity Data Journal.

[B4207585] Wirta H., Várkonyi G., Rasmussen C., Kaartinen R., Schmidt N. M., Hebert P. D. N., Barták M., Blagoev G., Disney H., Ertl S., Gjelstrup P., Gwiazdowicz D. J., Huldén L., Ilmonen J., Jakovlev J., Jaschhof M., Kahanpää J., Kankaanpää T., Krogh P. H., Labbee R., Lettner C., Michelsen V., Nielsen S. A., Nielsen T. R., Paasivirta L., Pedersen S., Pohjoismäki J., Salmela J., Vilkamaa P., Väre H., Tschirnhaus M. von, Roslin T. (2015). Establishing a community-wide DNA barcode library as a new tool for arctic research. Molecular Ecology Resources.

